# An evolutionary computational theory of prefrontal executive function in decision-making

**DOI:** 10.1098/rstb.2013.0474

**Published:** 2014-11-05

**Authors:** Etienne Koechlin

**Affiliations:** Institut National de la Santé et de la Recherche Médicale, Université Pierre et Marie Curie, Ecole Normale Supérieure, 29 rue d'Ulm, 75005 Paris, France

**Keywords:** prefrontal cortex, executive control, decision-making, reasoning, Bayesian inference, reinforcement learning

## Abstract

The prefrontal cortex subserves executive control and decision-making, that is, the coordination and selection of thoughts and actions in the service of adaptive behaviour. We present here a computational theory describing the evolution of the prefrontal cortex from rodents to humans as gradually adding new inferential Bayesian capabilities for dealing with a computationally intractable decision problem: exploring and learning new behavioural strategies versus exploiting and adjusting previously learned ones through reinforcement learning (RL). We provide a principled account identifying three inferential steps optimizing this arbitration through the emergence of (i) factual reactive inferences in paralimbic prefrontal regions in rodents; (ii) factual proactive inferences in lateral prefrontal regions in primates and (iii) counterfactual reactive and proactive inferences in human frontopolar regions. The theory clarifies the integration of model-free and model-based RL through the notion of strategy creation. The theory also shows that counterfactual inferences in humans yield to the notion of hypothesis testing, a critical reasoning ability for approximating optimal adaptive processes and presumably endowing humans with a qualitative evolutionary advantage in adaptive behaviour.

## 1. Introduction

The prefrontal cortex subserves executive control and decision-making for coordinating and selecting thoughts and actions in the service of adaptive behaviour. Present in all mammals [1], the prefrontal cortex in rodents mainly reduces to paralimbic brain regions including the orbitofrontal cortex (OFC) and anterior-cingulate cortex (ACC) [1]. In primates, the prefrontal cortex has evolved with the development of lateral prefrontal regions (LPC) [2]. In humans, the LPC has further evolved with the emergence of the left–right asymmetry yielding to the notion of Broca's area [3,4] subserving human language [5] and bilaterally, in its most anterior portion, a polar region [6,7] (lateral frontopolar cortex, lFPC) which apparently has no homologues in monkeys [8,9] and subserves human reasoning [10].

The prefrontal cortex forms loop circuits with basal ganglia. These subcortical brain nuclei are common to vertebrates and include especially the striatum, which subserves reinforcement learning (RL) [11–14]. RL and, more specifically, temporal-difference RL algorithms are basic online adaptive processes that adjust a behavioural strategy mapping stimuli onto actions according to the discrepancy between actual and expected rewards. Importantly, RL is both a very simple and robust adaptive process that can learn a variety of complex tasks even in uncertain environments. In particular, when rewards only depend upon current states and actions and each state is encountered sufficiently often, RL converges towards the behavioural strategy maximizing rewards [15]. Evidence in rodents, primates and humans indicates that the ventral striatum processes reinforcing signals such as reward prediction errors that serve to adjust stimulus–response associations, whereas the dorsal striatum in relation to the premotor cortex processes stimulus–response associations guiding action selection [13,16–18].

However, RL has severe adaptive limitations. The most evident and crucial limitation is that learning new behavioural strategies erases previously learned ones. Indeed, the ability to store and re-use previously learned strategies confers an evolutionary advantage in environments exhibiting external contingencies that change and reoccur periodically (i.e. recurrent situations). In open-ended environments, however, where additionally new external contingencies may always appear, arbitrating between exploring/learning new behavioural strategies versus exploiting/adjusting previously learned ones raises an intractable computational problem. Here, we propose a computational theory postulating that the prefrontal cortex has evolved as primary solving this arbitration problem.

The statistically optimal solution involving Dirichlet processes mixtures [19] is computationally intractable for the two following reasons. First, arbitrating between creating new strategies versus adjusting previously learned ones is in essence non-parametric. This requires optimal adaptive processes to systematically re-evaluate offline past arbitrations whenever new information is acquired and consequently, to revise the repertoire of previously learned strategies in a backward fashion. Second, optimal adaptive processes require monitoring online the whole repertoire of learned strategies that continuously increase when new strategies are created. These computational requirements for optimal arbitrations rapidly yield to intractable computations, suggesting that the prefrontal cortex has evolved as implementing online only forward inferences over a small portion of the repertoire of learned strategies. Accordingly, our theory postulates that under these constraints, the development of prefrontal regions from lower mammalians to humans gradually adds new inferential/computational capabilities beyond RL, which increasingly optimize the arbitration between exploring/learning new behavioural strategies versus exploiting/adjusting previously learned ones. Assuming that basal ganglia implement the ongoing strategy that adjusts to external contingencies through RL and guides behaviour (referred to as *the actor* strategy or simply *the actor*), the prefrontal cortex may have evolved as arbitrating online between these two options: (i) staying with the current actor strategy, which adjusts through RL to external contingencies and (ii) switching away from the current actor strategy and creating a new one from the previously learned strategies stored in long-term memory for driving subsequent behaviour.

## 2. Reactive inferences, memory recollection and the paralimbic prefrontal cortex

Arbitrating between these two options first requires inferring when external contingencies change and require switching away from the current actor strategy. Our theory assumes that the development of paralimbic prefrontal regions in lower mammals implements a first inferential step: namely, inferring such changes based on the inconsistency between actual action outcomes and the outcome contingencies the actor has learned so far. Such inferences are *factual* as they only bear upon the outcome predictive model the actor strategy has learned, and *reactive* as they operate only after observing action outcomes.

**Figure 1.**
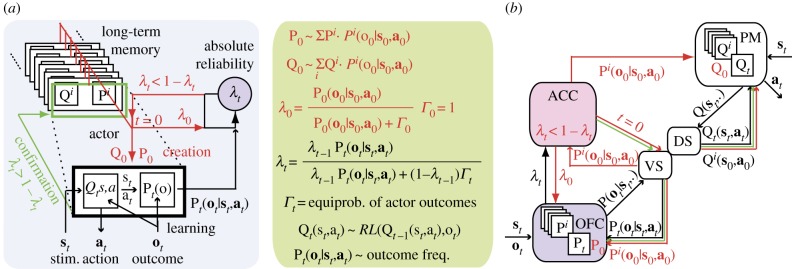
Factual reactive inferences in the rodent prefrontal cortex. (*a*) Inferential system arbitrating between actor learning and creation from long-term memory. Q, selective and P, predictive models forming behavioural strategies stored in the long-term memory repertoire (superscript ***i***). Subscript ***t*** (trial number) indicates actor strategy driving ongoing behaviour and learning external contingencies through reinforcement learning (RL) and action outcome frequencies. *λ_t_*, actor absolute reliability inferred online (right inset: ***Γ****_t_*, default-likelihood). *t* = 0, time when the actor becomes unreliable (*λ_t_* < 1 − *λ_t_*) and a new actor is created by mixing stored strategies weighted according to predictive models. Green, confirmation events when the actor becomes reliable (*λ_t_* > 1 − *λ_t_*). (*b*) Presumed system implementation in paralimbic prefrontal regions (rodents). DS, dorsal and VS, ventral striatum filtering out non-actor strategies and learning external contingencies. PM, ACC and OFC, premotor, anterior-cingulate cortex and orbitofrontal cortex, respectively. Red, actor creation triggered in ACC; actor filtering in the striatum is off and allows mixing strategies stored in OFC and PM. See text for explanation.

To make such inferences, the actor thus learns both a *selective* and *predictive* model: the former maps stimuli onto actions, adjusts through RL and enables selection of the most rewarding actions in response to stimuli; the latter maps stimulus–action associations onto expected outcomes and learns by simply registering outcome frequencies given responses to stimuli. Critically, the predictive model enables inference online of the actor *absolute reliability*, i.e. the posterior probability the current external contingencies match those the actor has learned (figure 1*a*). Updating online actor absolute reliability according to actual action outcomes involves forward Bayesian inferences and requires comparison of the likelihood of actual action outcomes derived from the actor predictive model with their likelihood according to *any* potential alternative models. The latter cannot be exactly computed, because the range of possible external contingencies is presumably infinite and unknown. However, that likelihood can be estimated as reflecting the maximal predictive entropy, namely as the equiprobability of action outcomes *produced by* the actor. We referred to this estimate as the *default outcome-likelihood*.

Actor absolute reliability *λ_t_* in every trial *t* serves to arbitrate between staying versus switching from the current actor strategy. When the actor remains more likely reliable than unreliable (*λ_t_* > 1 − *λ_t_*), no changes in external contingencies are likely to have occurred. The same actor strategy is then kept and continues to adjust through RL. The system thus operates in an *exploitation* mode. When conversely the actor becomes unreliable (*λ_t_* < 1 − *λ_t_*), external contingencies have likely changed. A new actor is then built from optimally using the whole repertoire of previously learned strategies stored in long-term memory, which correspond to the former actor strategies (including the one that has just become unreliable). The new actor selective (predictive, resp.) model is thus formed as the mixture of selective (predictive, resp.) models stored in the repertoire possibly weighted according to current action outcome given strategies' predictive models (figure 1*a*).

In this mixture process, importantly, selective models may be recalibrated according to current rewarding values of action outcomes through a *model-based* RL process [20]: selective and predictive models serve as action and outcome predictors, respectively, for implementing covert RL, whereby outcome rewarding values are possibly altered with respect to current animal needs (e.g. satiety effects). This model-based RL process calibrates the new actor selective model according to current animal needs.

As the new actor is created from the strategies repertoire, its initial absolute reliability corresponds to the repertoire absolute reliability, i.e. the probability the current external contingencies match those associated with one stored strategy, or equivalently, the probability that the animal faces a previously encountered situation. This absolute reliability is inferred using Bayes' law, which requires evaluating the likelihood of the current action outcome in every previously encountered situation and separately, in any possible new situations (figure 1, inset). In the former case, this likelihood is simply derived from the mixture of stored predictive models (corresponding to the new actor predictive model). In the latter case, the likelihood is estimated as the default outcome-likelihood associated with the presumably new situation. It is actually equal to 1, because in this situation, only one outcome has been observed. As a result, the new actor guiding behaviour is initially inferred as being unreliable (figure 1, inset). The system thus operates in an *exploration* mode that promotes actor learning by preventing switching again, *while* the actor remains unreliable. When the actor becomes reliable, the system returns to the exploitation mode, whereby switching away from the actor strategy (when it again becomes unreliable) and creating a new one may occur again. Note that the lower the initial actor reliability is, the longer exploration will last: initially, the new actor less likely matches the new situation and, consistently, more trials are required for the new actor to learn the new external contingencies.

Under its intrinsic computational constraints (forward, factual and reactive inferences only), this model is an optimal adaptive system in environments featuring both new and recurrent situations. The model especially exhibits three key functional properties, which are consistent with empirical data. First, the model shows abrupt rather than gradual behavioural changes, when following variations in external contingencies, the actor strategy becomes unreliable, and a new one is created. Such abrupt behavioural changes are routinely observed in rodents, primates and human experiments [21–24]. Second, the long-term repertoire of behavioural strategies expands whenever new actors are created, so that the *reoccurrence* frequencies of external situations have a major influence on shaping new actors. Whenever external situations reoccur, new actors are created with selective and predictive models learning again the associated external contingencies, thereby replicating in the repertoire the selective and predictive models previously learned from previous occurrences: the more external situations reoccur, the more these models are then replicated in long-term memory. Consequently, external contingencies learned from situations that more frequently reoccur contribute more to the formation of new actor strategies. This computational model thus exhibits a basic feature of Dirichlet processes [19]. Collins & Koechlin [25] showed that the model accounts for increasing human performances associated with recurrent situations. Third, actor creation involves model-based RL, whenever ongoing actors driven by standard (model-free) RL become unreliable. Arbitrating between adjusting the current actor versus creating a new one thus yields to a decision between model-based and model-free RL, which accounts for behavioural changes observed in rodents (e.g. extinction effects) following outcome devaluation manipulations [20].

As generally agreed, the premotor cortex along with the dorsal striatum encodes and stores selective models of behavioural strategies [26–28], whereas the dorsal and ventral striatum implement RL adjusting the actor selective model (see above). Our hypothesis is that operating beyond RL, the factual and reactive inferential system is implemented in paralimbic prefrontal regions (figure 1, right). The OFC (especially the medial OFC in humans) encodes strategies' predictive models and updates actor absolute reliability according to action outcomes. Empirical data in rodents, monkeys and humans show that, consistently, this region responds to action outcomes in relation with outcome predictions [29–33]. Moreover, human neuroimaging studies showed that the medial OFC is involved in monitoring the ongoing course of action [34] and inferring changes in external contingencies [33].

By contrast, the ACC is assumed to detect when the actor becomes unreliable for triggering the creation of new actors. Rodent studies show that in the ACC, neuronal activity consistently exhibits abrupt phase transitions in relation to behavioural switches [24]. Moreover, monkey electrophysiological and human neuroimaging studies indicate that the ACC is involved in monitoring when to switch from exploitation to exploration behaviours [35,36], whereas adjacent medial (pre)supplementary motor regions are involved in inhibiting established behavioural responses [37] and promoting exploratory responses [38]. More generally, the ACC is involved in responding to surprising outcomes triggering behavioural switches [39] and in starting the execution of new tasks [40].

Empirical data (review in [41]) further suggest that prefrontal–striatal loop circuits involving the ACC and OFC [42] may subserve actor creation. The ACC may prevent the striatum from filtering out non-actor strategies and allow stored strategies to mix for forming new actors: the ventral striatum, which for every stored strategy then receives the outcome-likelihood from the OFC, may return these likelihoods to both the ACC and OFC; assuming that the ACC further conveys these pieces of information to the premotor cortex, the mixture of selective and predictive models given current action outcomes may then occur in the premotor cortex and OFC, respectively. The ACC may concomitantly initialize actor reliability.

The theory indicates that new actors are initially unreliable (exploration mode) and when they become reliable, the system returns to the exploitation mode. This event may correspond to an internal reinforcing signal consolidating new actor selective and predictive models in long-term memory. This event may thus be detected in the ventral striatum, which processes behavioural reinforcers and receives projections from the OFC presumably inferring actor reliability. The theory thus predicts that the ACC triggers exploration, whereas the ventral striatum signals when to return to exploitation.

## 3. Proactive inferences and contextual control in the lateral prefrontal cortex

The above-described inferential system has two critical limitations. First, its adaptive capability is only reactive: new actor strategies are created only after experiencing action outcomes, which may be detrimental with adverse outcomes. Second, actor creation ignores the context in which stored strategies were learned. Accordingly, our theory assumes that the development of LPCs in primates implements a second inferential step overcoming these limitations: namely, further inferring from external cues when to switch away from the actor strategy. In contrast to action outcomes, external cues occur independently of subjects' behaviour but their occurrences may also inform about changes in external contingencies. The resulting inferential system thus exhibits *proactive* behaviours, because external cues typically alter the arbitration between adjusting versus creating actor strategies before acting.

**Figure 2.**
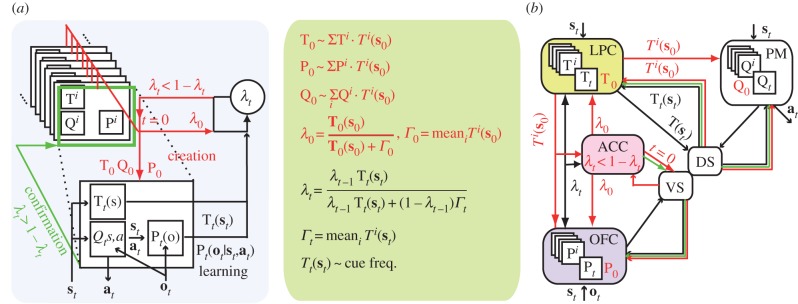
Factual reactive and proactive inferences in the primate prefrontal cortex. (*a*) Same inferential system shown in figure 1 but adding proactive inferences arbitrating between actor learning and creation according to current external cues (red, actor creation following proactive inferences). T, contextual models learning likelihoods of external cues associated with strategies and allowing revising actor absolute reliability before acting (inset). (*b*) Presumed implementation of proactive inferences in lateral prefrontal regions (LPC, primates) in addition to reactive inferences in OFC shown in figure 1. Detailed legend in figure 1.

For making such proactive inferences, the actor strategy learns an additional internal model, which we refer to as the *contextual model*. The actor contextual model simply registers the frequencies of external cues and is stored in long-term memory along with selective and predictive models (figure 2). When external cues occur, the actor absolute reliability is then updated through forward Bayesian inference, which requires comparison of the likelihood of these external cues derived from the actor contextual model with their likelihood according to *any* potential alternative models. The latter is again not exactly computable. External cues are however independent of subjects' behaviour. This likelihood can therefore be estimated as the frequency of current external cues observed in the past, which simply derives from the mean of contextual models stored in long-term memory. This estimate is referred to as the *default context-likelihood.* Consequently*,* external cues that are less likely to occur in the current than past situations, degrade the actor absolute reliability and may yield to proactively switch away from the current actor strategy for creating a new one.

Whenever new actors are created following reactive and/or proactive inferences, their selective (predictive and contextual, respectively) model is again computed as the mixture of selective (predictive and contextual, resp.) models stored in long-term memory. The mixture however is now weighted according to current action outcomes and/or external cues given predictive and/or contextual models (figure 2). Accordingly, actor creation may now depend upon current external cues along with action outcomes and the frequency of recurrent situations. In particular, strategies learned within more distinct contexts than the current one contribute less to actor creation.

New actor initial reliability again corresponds to the repertoire absolute reliability, but the latter is now evaluated according to current action outcomes and external cues (figure 2, inset). This absolute reliability is computed as above based on new actor predictive and contextual models in relation to the default outcome- and context-likelihood. Importantly, new actor strategies may now be formed as being immediately reliable; this happens when current external cues match those under which strategies already in the repertoire were learned. In that event, new actor strategies may then be rejected as soon as they serve as actor according to subsequent cue- and outcome-based counterevidence. Proactive inferences thus provide the ability to control behaviour according to the context, i.e. to rapidly recreate and switch across behavioural strategies according to external cues.

Under its intrinsic computational constraints (forward and factual inferences only), this computational model optimally uses external cues and action outcomes for adapting to environments featuring both new and recurrent situations. Our hypothesis is that the lateral prefrontal cortex (LPC) learns and encodes contextual models and updates the actor absolute reliability according to external cues (figure 2). Monkey and human studies show that, consistently, the LPC subserves the formation and selection of behavioural strategies according to contextual cues [43–45]. The neuronal connections between the LPC, OFC and premotor cortex [46] are further assumed to link the contextual, predictive and selective models associated with the same strategy. More specifically, the LPC revises the actor absolute reliability conveyed from the OFC according to external cues and returns the updated reliability to the OFC (and vice versa). The ACC again detects when the actor becomes unreliable for triggering actor creation.

Actor creation requires mixing of selective (predictive and contextual, resp.) models over stored strategies according to outcome and cue likelihoods. This may be achieved as described above through the architecture of cortical–cortical and striatal–cortical connections within the frontal lobes [42,46] (figure 2). The ACC prevents the striatum from filtering out non-actor strategies, so that for every strategy, the ventral striatum returns the outcome-likelihood to the OFC and ACC, whereas the dorsal striatum returns the cue likelihood to the LPC. The new actor predictive model requires mixing of predictive models, which may occur in the OFC through LPC-to-OFC projections conveying cue likelihoods. The new actor contextual model requires mixing of contextual models, which may occur in the LPC through ACC-to-LPC projections conveying outcome likelihoods. Finally, the new actor selective model requires mixing of selective models, which may occur in the premotor cortex through LPC-to-premotor projections conveying both outcome and cue likelihoods.

## 4. Counterfactual inferences, hypothesis testing and the frontopolar cortex

In the above-described inferential system, the critical limitation is that inferences remain factual: the decision to adjust versus change the ongoing behavioural strategy, i.e*. the actor*, bears upon the actor reliability only. Accordingly, the theory assumes that the development of the frontopolar cortex (lFPC) in humans implements a third inferential step overcoming this limitation: namely, inferring when to change the current actor strategy from concurrently monitoring the reliability of multiple behavioural strategies. The human executive system thus develops counterfactual inferences bearing upon alternative behavioural strategies that are not guiding ongoing behaviour. These counterfactual inferences enable the inference online not only of *when* to change the actor strategy, but also *which* strategy may replace it. Ideally, counterfactual inferences should bear upon the whole repertoire of stored strategies. This seems however computationally costly and biologically implausible. Our theory therefore assumes that counterfactual inferences develop only over a limited number of stored strategies, referred to as the inferential buffer.

One might consider the inferential buffer as forming a global actor strategy, whereby action selection and strategy learning result from mixing online monitored strategies over the buffer according to their relative reliability [47]. Collins & Koechlin [25] showed that this view is inconsistent with human behavioural performances in sequential decision tasks. This is also theoretically problematic, because the global actor may be inferred as being reliable with only unreliable strategies: the mixture of monitored strategies may therefore be strongly suboptimal when another strategy stored in long-term memory is potentially reliable. More optimally, the executive system may concurrently infer the absolute reliability of every monitored strategy and when none are inferred as being reliable, a new strategy is created from long-term memory (as described above) and added to the inferential buffer. When, conversely, one is inferred as being reliable, the others are necessary unreliable, even when considered collectively (absolute reliabilities sum up to 1 or less). Accordingly, the reliable strategy becomes *the* actor strategy selecting actions and learning external contingencies (i.e. adjusting its selective, predictive and contextual models). The buffer is thus assumed to comprise the actor strategy driving behaviour plus a number of alternative strategies, which for clarity we refer to as *counterfactual* strategies.

**Figure 3.**
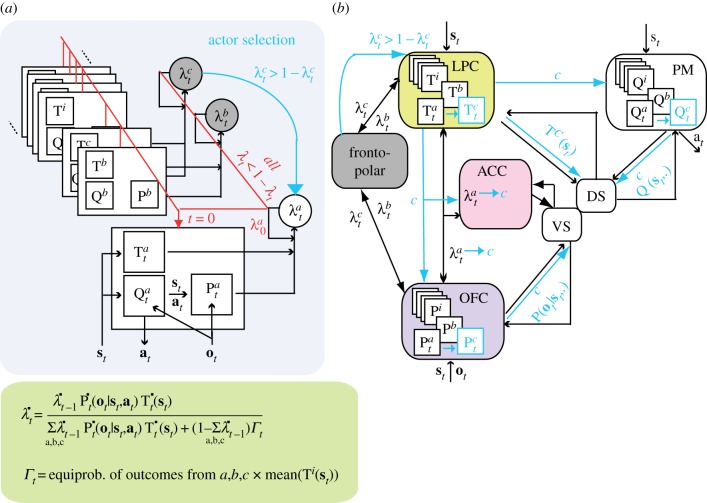
Counterfactual inferences in the human prefrontal cortex. (*a*) Same inferential system shown in figure 2 but adding counterfactual inferences (grey) for arbitrating between actor learning, switching and creation. Superscript a, b, c: actor (a) and two additional counterfactual (b,c) strategies, which absolute reliability is concurrently inferred online (bottom inset, equations show combined proactive and reactive inferences for every monitored strategy). Actor switching (blue, from a to c in the shown example) occurs when one counterfactual strategy becomes reliable. Actor creation (red) occurs when all monitored strategies become unreliable. (*b*) Presumed implementation of counterfactual inferences through the human frontopolar cortex, LPC (proactive component) and OFC (reactive component). The frontopolar cortex encodes counterfactual absolute reliabilities, while the OFC encode actor reliability. Actor switching presumably originates from the LPC and diffuses in the prefrontal network in a top-down fashion (blue arrows). Actor creation is not shown for clarity (identical to figures 1 and 2). Detailed legend in figure 1.

The actor strategy may thus be changed rather than adjusted through RL by either *retrieving* and *switching to* a reliable counterfactual strategy or *creating* a new strategy from long-term memory (figure 3*a*). In the former case, the system operates in the *exploitation* mode, because the new actor remains reliable. In the latter case, the new actor may be created as being unreliable; in that case the inferential system switches into the *exploration* mode. The system subsequently returns to the exploitation mode in two ways. Either a counterfactual strategy becomes reliable, while the newly created actor remains unreliable. The former then becomes the actor, and the latter is *rejected* from the buffer and disbanded. Or the newly created actor becomes reliable, whereas the counterfactual strategies remain unreliable. The former is then *confirmed* and stored in long-term memory along with others. Exploration periods thus correspond to *hypothesis testing* on strategy creation. Accordingly, counterfactual strategies are the former actors that have been reliably assigned to an external situation that previously occurred. When newly created actors are confirmed, however, the number of strategies monitored in the buffer increases and possibly reaches its capacity limit. In that event, the theory assumes that the strategy used the least recently as actor is discarded from the buffer. The rationale is that older situations are less frequent and less likely to reoccur in the short-run. The buffer therefore keeps monitoring counterfactual strategies which will more likely match the *next* external situation.

The computations implementing this counterfactual inferential system are essentially the same as those described above. Reactive and proactive inferences are simply extended to counterfactual strategies. The differences are as follows (figure 3, inset): first, absolute reliability is inferred for the actor and counterfactual strategies, so that the *default outcome-likelihood* is now better estimated as the equiprobability of outcomes produced by the actor *and* counterfactual strategies. Second, the absolute reliability of actor and counterfactual strategies directly weights their relative contribution in creating new actors from long-term memory. Collins & Koechlin [25] showed that this computational algorithm predicts human choices in recurrent and new situations featuring uncertain and variable contingencies possibly associated with the occurrences of contextual cues. Moreover, all the model components appeared necessary for accounting for human performances. The best account was found when the buffer capacity corresponds to two/three counterfactual strategies. This size matches the capacity previously proposed for human (declarative) working memory [48].

The hypothesis is that the lFPC encodes the absolute reliability of counterfactual strategies (figure 3*b*). Consistently, neuroimaging studies show that the lFPC is engaged in cognitive branching, that is, holding on the execution of one task during the performance of another task [10,49] and in monitoring the opportunity to switch to alternative courses of action [34]. The lFPC has also major reciprocal connections with the OFC and LPC [9,46]. Accordingly, the (medial) OFC may update counterfactual strategies' reliabilities encoded in the lFPC according to action outcomes (given predictive models presumably stored in the OFC). Similarly, the LPC may update them according to external cues (given contextual models presumably encoded in the LPC).

In contrast to actor creation, retrieving counterfactual strategies as new actors when they become reliable requires *selecting* and reactivating the corresponding selective, predictive and contextual models from long-term memory to drive behaviour. The LPC may be the best candidate for this function (figure 3*b*). Adjacent to the lFPC, the LPC is the only prefrontal region strongly connected to both the premotor cortex and OFC, presumably storing selective and predictive models, respectively [46]. Thus, selecting a reliable contextual model in the LPC may concomitantly induce the selection of associated models in the premotor cortex and OFC. Consistent with the hypothesis, the LPC is involved in retrieving action sets through top-down selection from LPC to premotor regions [44,50].

## 5. Discussion

We propose here that the prefrontal cortex has primary evolved from lower mammalians to humans by gradually adding new inferential capabilities beyond RL, which progressively optimize the arbitration between exploring/learning new behavioural strategies versus exploiting/adjusting previously learned ones. This arbitration is optimized assuming that the environment varies according to both recurrent and new causes, which are indirectly observable, independent and potentially infinite. Optimization has occurred under the computational constraint that the brain implements only forward, online inferential processes bearing upon a limited portion of behavioural strategies stored in long-term memory.

With these assumptions, optimal arbitration is based on inferring online the *absolute reliability* of every monitored strategy, that is, the posterior probability that given external evidence, the current external contingencies match those the strategy has learned. Monitored strategies may therefore be inferred as being reliable (more likely matching than differing) versus unreliable (the converse). When one is reliable, the others are individually *and* collectively unreliable. We indicate a solution indicating how the brain may compute absolute reliabilities through estimates of *default-likelihoods*, that is, likelihoods of external cues and action outcomes when the current external contingencies presumably match no monitored strategies. The concepts of default-likelihood and absolute reliability generalize the notion of expected/unexpected uncertainty proposed by Yu & Dayan [51] and provide the computational foundations of the present theory. They may be related to the psychological notions of metacognitive processes and confidence judgements [52].

Based on this computational framework, we identify three critical inferential capabilities associated with the development of specific prefrontal regions. The OFC and ACC appearing in rodents provides the ability to make *factual*, *reactive* reliability inferences; reliability inferences are based on action outcomes and only bear upon the actor strategy guiding action and learning external contingencies. The OFC is predicted to encode strategies' internal models predicting action outcomes and to revise actor absolute reliability according to action outcomes. Critically, the actor strategy learns actions through RL so that the more outcomes are rewarding, the more the reliability reflects the predicted occurrence of these outcomes. The ACC, by contrast, detects when the actor strategy becomes unreliable for triggering the creation of new actor strategies from long-term memory. The LPC appearing in primates provides the additional ability to make *factual*, *proactive* inferences. The LPC is predicted to encode strategies' internal models predicting external cues and to revise actor absolute reliability according to external cues typically occurring before action. Finally, the lFPC appearing in humans provides the ability to further make both reactive and proactive *counterfactual* inferences. The lFPC is predicted to encode the absolute reliability of a few counterfactual strategies stored in long-term memory; along with actor reliability, counterfactual reliabilities are revised in OFC and LPC according to action outcomes and external cues, respectively.

Counterfactual strategies along with the actor strategy form an inferential/monitoring buffer essentially equivalent to the psychological notion of procedural working memory [48,53,54]. The notion of actor is consistent with the idea of attentional focus within working memory [53–55]. When one monitored strategy becomes reliable, this strategy becomes the actor. When the actor *becomes* unreliable with no reliable counterfactual alternatives in the buffer, a new actor is created from long-term memory. Actor creation consists of optimally mixing strategies stored in long-term memory according to current outcome-based and/or cue-based evidence. Actor creation is thus a model-based construct of actor strategies involving model-based RL [20]. The theory predicts that this model-based construct of actors occurs through striatal–frontal loop circuits. As newly created actors subsequently adjust through model-free RL, the actor strategy gradually results from the hybridation between model-based and model-free RL, whereby model-free RL progressively dominates with time. This hybridation resulting from abrupt and intermittent model-based constructs of new actors, when model-free RL adjustments of ongoing actors become unreliable, is optimal with forward inferential processes operating online and bearing upon a limited number of counterfactual strategies.

Newly created actors may be inferred as being initially unreliable indicating that the environment is likely in a state that was not previously observed. The prefrontal executive system then switches into an exploration period corresponding to *hypothesis testing*: this unreliable actor guides behaviour and may be subsequently confirmed or rejected. Confirmation occurs when this actor become reliable before any counterfactual strategies. The ventral striatum is predicted to detect this confirmation event yielding to the actor consolidation in long-term memory. Conversely, rejection occurs when a counterfactual strategy become reliable before this actor. The LPC is predicted to retrieve this reliable counterfactual strategy to serve as actor. Critically, hypothesis testing prevents the capacity-limited buffer from monitoring unnecessary strategies emerging from long-term memory. Hypothesis testing, moreover, is a primitive form of backward inferences, because every decision to create new strategies may be subsequently revised on the basis of subsequent information. Backward inferences are actually critical in optimal adaptive systems operating in open-ended environments for dealing with the non-parametric nature of strategy creation [19]. Thus, counterfactual inferences and hypothesis testing associated with the development of the lFPC appear as critical reasoning capabilities endowing humans with a qualitative evolutionary advantage in adaptive behaviour.

The present theory provides a unified, principled model of the overall inferential architecture of the human prefrontal cortex. The model makes testable predictions outlined above [56]. This overall model, however, masks the computational complexity of neuronal processing involved in learning strategies' internal models coding the likelihoods of action outcomes and external cues in the OFC and LPC. Indeed, action outcomes and external cues may vary within high-dimensional, continuous spaces possibly including the time dimension. Accordingly, the OFC and LPC regions are likely to operate in lower-dimensional spaces through categorization and extra/interpolation processes based on relative space metrics [57] for representing action outcomes and external cues and computing their likelihood in relation to behavioural strategies. As recently proposed [57], these coding processes may have concurrently emerged through the development of multiple subregions in OFC and LPC in association with posterior associative cortical regions.
